# Improving the annotation of the cattle genome by annotating transcription start sites in a diverse set of tissues and populations using Cap Analysis Gene Expression sequencing

**DOI:** 10.1093/g3journal/jkad108

**Published:** 2023-05-22

**Authors:** Mazdak Salavati, Richard Clark, Doreen Becker, Christa Kühn, Graham Plastow, Sébastien Dupont, Gabriel Costa Monteiro Moreira, Carole Charlier, Emily Louise Clark

**Affiliations:** The Roslin Institute, University of Edinburgh, Edinburgh EH25 9RG, UK; Edinburgh Clinical Research Facility, Genetics Core, University of Edinburgh, Edinburgh EH4 2XU, UK; Institute of Genome Biology, Research Institute for Farm Animal Biology (FBN), Dummerstorf 18196, Germany; Institute of Genome Biology, Research Institute for Farm Animal Biology (FBN), Dummerstorf 18196, Germany; Faculty of Agricultural and Environmental Sciences, University Rostock, Rostock 18059, Germany; Department of Agricultural, Food and Nutritional Science, Livestock Gentec, University of Alberta, Edmonton T6G 2H1, Canada; Unit of Animal Genomics, GIGA Institute, University of Liège, Liège 4000, Belgium; Unit of Animal Genomics, GIGA Institute, University of Liège, Liège 4000, Belgium; Unit of Animal Genomics, GIGA Institute, University of Liège, Liège 4000, Belgium; Faculty of Veterinary Medicine, University of Liège, Liège 4000, Belgium; The Roslin Institute, University of Edinburgh, Edinburgh EH25 9RG, UK

**Keywords:** transcription start site, CAGE-Seq, cattle genome, BovReg, FAANG, EuroFAANG

## Abstract

Understanding the genomic control of tissue-specific gene expression and regulation can help to inform the application of genomic technologies in farm animal breeding programs. The fine mapping of promoters [transcription start sites (TSS)] and enhancers (divergent amplifying segments of the genome local to TSS) in different populations of cattle across a wide diversity of tissues provides information to locate and understand the genomic drivers of breed- and tissue-specific characteristics. To this aim, we used Cap Analysis Gene Expression (CAGE) sequencing, of 24 different tissues from 3 populations of cattle, to define TSS and their coexpressed short-range enhancers (<1 kb) in the ARS-UCD1.2_Btau5.0.1Y reference genome (1000bulls run9) and analyzed tissue and population specificity of expressed promoters. We identified 51,295 TSS and 2,328 TSS-Enhancer regions shared across the 3 populations (dairy, beef-dairy cross, and Canadian Kinsella composite cattle from 2 individuals, 1 of each sex, per population). Cross-species comparative analysis of CAGE data from 7 other species, including sheep, revealed a set of TSS and TSS-Enhancers that were specific to cattle. The CAGE data set will be combined with other transcriptomic information for the same tissues to create a new high-resolution map of transcript diversity across tissues and populations in cattle for the BovReg project. Here we provide the CAGE data set and annotation tracks for TSS and TSS-Enhancers in the cattle genome. This new annotation information will improve our understanding of the drivers of gene expression and regulation in cattle and help to inform the application of genomic technologies in breeding programs.

## Introduction

Genomic technologies are used widely and successfully in breeding programs for cattle, and other farmed animal species, across the globe, to improve health, welfare, and productivity (Van Eenennaam *et al*. 2014). The success of applying genomic technologies depends considerably on the quality of the reference genome for each species. For domestic cattle, the current reference genome (ARS-UCD1.2) is one of the most contiguous, complete, and accurate reference genomes for a farmed animal species (Rosen *et al*. 2020). ARS-UCD1.2 was assembled from DNA sequence from a single inbred Hereford breed cow, L1 Dominette 01449, and provides a hugely valuable resource to inform cattle breeding (Rosen *et al*. 2020). With a highly accurate reference genome sequence now available for cattle, efforts have shifted towards annotating the ARS-UCD1.2 sequence to define the function of each genomic region (reviewed in Giuffra and Tuggle 2019).

Defining robust genomic annotations has proven to be useful in the sustained genetic improvement of farmed animals (Georges *et al*. 2019). High-resolution mapping of the actively transcribed regions of the genome can help to identify the genomic drivers of gene expression and regulation (Tippens *et al*. 2018; Guerrini *et al*. 2022). Defining transcription start sites (TSS) within promoter regions, for example, provides information about how genes are expressed and regulated across different tissues and cell types. Within TSS are transcription factor binding sites that control gene expression and integrate information from other *cis*-regulatory elements such as enhancers. Recently, the theory of multiple expression clusters within promoters has been used to annotate and fine map TSS and associated enhancers within mammalian transcriptomes (Frith *et al*. 2008; Andersson *et al*. 2014). These putative core promoter and associated enhancer regions are defined using 5′ cap transcript sequencing, e.g. via RNA Annotation and Mapping of Promoters for the Analysis of Gene Expression (RAMPAGE) (Batut *et al.* 2013) and Cap Analysis Gene Expression (CAGE) (Takahashi *et al*. 2012). RAMPAGE and CAGE have been used successfully to annotate TSS in cattle (Goszczynski *et al*. 2021; Ross *et al*. 2022), pig (Robert *et al*. 2015), sheep (Salavati *et al*. 2020), and other vertebrate species (Forrest *et al*. 2014; Robert *et al*. 2015; Deviatiiarov *et al*. 2017; Noguchi *et al*. 2017). These data sets can be integrated with quantitative trait loci (QTL) and omics data for comparative analyses and are very useful for interpretation of the effects of functional genetic variants at a genome-wide scale (reviewed in Guerrini *et al*. 2022).

The reference genome for domestic cattle, ARS-UCD1.2, now has a high-quality annotation of both expressed and regulatory regions, across many different tissue types, generated for the Hereford breed (Halstead *et al*. 2020; Goszczynski *et al*. 2021). Accurate annotation of the location of TSS, in particular, is essential for understanding the regulatory mechanisms that control gene expression. Goszczynski *et al*. (2021) used RAMPAGE sequencing to map TSS across 31 tissues from 2 male and 2 female adult Hereford cattle. The extent to which the location of TSS might differ across other ages, breeds, and populations of cattle remains poorly understood. This lack of knowledge hinders efforts to define and predict the effects of transcriptomic variation on breed- or population-specific characteristics, such as fertility or milk yield. Generating transcriptomic data sets that capture transcriptional complexity across multiple breeds or populations will help to address this knowledge gap. For example, transcriptomic resources generated by Ross *et al*. (2022) revealed a large amount of transcriptional variation in fertility genes in Brahman (*Bos taurus indicus*) cattle.

In this study, we use CAGE sequencing (Takahashi *et al*. 2012) to precisely define TSS across a set of 24 tissues from 3 different populations of cattle: dairy [Belgian Holstein Friesian (HOL)], beef–dairy cross (German Charolais × Holstein F2), and Canadian Kinsella cattle (beef composite). By including both beef and dairy populations, we will provide a transcriptomic resource that could be used to identify functional genomic features affecting selected or adapted traits in both production types (Halstead *et al*. 2020; Alexandre *et al*. 2021). Several transcriptomic data sets (RNA-Seq and small RNA-Seq) are being generated from the same set of 24 tissues, as part of a wider effort in the EU H2020 BovReg project (https://eurofaang.eu/projects/bovreg). One of the main aims of the BovReg project is to generate a high-resolution transcriptomic map to improve the annotation of the ARS-UCD1.2 reference assembly, by adding transcriptomic information for multiple tissue samples across the 3 different ontogenetic ages and populations (Moreira *et al*. 2022). This additional annotation information will improve our understanding of the drivers of gene expression and promoter variety/plasticity in cattle and help to inform the application of genomic technologies in breeding programs.

## Materials and methods

### Animals

Twenty-four tissue samples from each of 3 different cattle populations, which had been collected for previous studies, were chosen for the purpose of this study. The 3 populations were dairy (HOL), beef–dairy cross (Charolais × Holstein F2), and Canadian Kinsella (beef composite). In the Canadian Kinsella composite (KC) population, Angus, Hereford, and Gelbvieh breeds account for ∼65% of the breed composition with signals from 9 other cattle breeds including Brown Swiss, Limousin, Simmental, Holstein, and Jersey. For each population, tissues had been collected from 2 animals (1 male and 1 female per population = 6 animals in total). These 6 animals included 3 different age groups (*neonatal*, *juvenile*, and *adult*). *Neonatal* includes HOL calves from Belgium (male calf 24 days and female calf 22 days), *juvenile* includes Canadian Kinsella steer (bullock 217 days) and Canadian Kinsella heifer (210 days) from Canada, and *adult* includes Charolais × Holstein F2 cow and bull (bull 18 months and cow 3 years, 7 months, and 13 days) from Germany. The Canadian and German animals were euthanized by captive bolt then exsanguination. Belgian calves were euthanized by intravenous administration of T-61 (embutramide 200 mg/mL, mebezonium iodure 50 mg/mL, and tetracaine chlorhydrate 5 mg/mL) directly followed by exsanguination. Post mortem dissection at all 3 geographical locations was supervised by a veterinarian. The animal experiments were performed in the same way across all 3 geographical locations. Details for the German animals, which were used as a guide to coordinate sample collection at the other 2 geographical locations, are included in Nolte *et al*. (2020, 2022) and in the protocol which is available via the FAANG data portal https://www.fbn-dummerstorf.de/fileadmin/media/I3.0/FBN_GenomePhysiology_SOP_CryofreezingTissueSsamples_20160331.pdf. Tissue samples for each of the 3 populations were snap frozen immediately upon collection and stored at −80°C for downstream RNA extraction.

The tissue samples prioritized for this study were chosen to represent 5 major organ systems: cardiopulmonary, central nervous, gastrointestinal (GI), immune, and urogenital. A sixth class of organ system termed “miscellaneous” included skeletal muscle, thyroid, and subcutaneous fat. Details of which tissue samples were analyzed are included in column 1 of Table 1. Due to the limitations of tissue sample collection, there are some instances where a tissue sample was not collected for 1 or more of the animals and these are indicated as “Tissue not available” in Table 1.

**Table 1.** List of all of the RNA samples with CAGE data generated for this study. The RNA samples for which CAGE libraries were generated and included in downstream analysis are indicated with “Yes.” Tissue samples that were not collected, or not available, from each of the animals from the 3 populations are indicated as “Tissue not available.” A small number of CAGE libraries (7 in total) had to be excluded from downstream analysis for other reasons which are indicated in the footnote to the table and further described in the *Results* section.

	Belgium		Germany		Canada	
	Neonatal		Adult		Juvenile	
	Dairy—HF		Beef–dairy—Char × Hol		Kinsella—KC	
Tissue	Male	Female	Male	Female	Male	Female
Adrenal gland cortex	Yes	Yes	Yes	Yes	Yes	*^a^* Library Excluded
Cerebellum	Yes	Yes	Yes	Tissue not available	Tissue not available	Yes
Cerebrum cortex	Yes	Yes	Yes	Yes	Yes	Tissue not available
Colon	Yes	Yes	Yes	Yes	Yes	Yes
Duodenum	Yes	Yes	Yes	Yes	Yes	Yes
Heart	Yes	Yes	Yes	Tissue not available	Yes	Yes
Hypothalamus	Yes	Yes	Tissue not available	Tissue not available	Tissue not available	Tissue not available
Ileum	Yes	Yes	Yes	Yes	Yes	Yes
Jejunum	Yes	Yes	Yes	Yes	Yes	Yes
Kidney	Yes	*^a^* Library excluded	Yes	Yes	Yes	Yes
Liver	Yes	YES	Yes	Yes	Yes	Yes
Lung	Yes	*^a^* Library excluded	Yes	Yes	Yes	Yes
Lymph node	Yes	Yes	Yes	Yes	Yes	Yes
Mammary gland	N/A sex-specific	Yes	N/A sex-specific	Yes	N/A sex-specific	Yes
Ovary	N/A sex-specific	Yes	N/A sex-specific	Tissue not available	N/A sex-specific	Yes
Pancreas	Yes	Yes	*^b^* Library excluded	*^b^* Library excluded	*^b^* Library excluded	*^b^* Library excluded
Pituitary gland	Tissue not available	Yes	Tissue not available	Tissue not available	Tissue not available	Tissue not available
Rumen	Yes	Yes	Yes	Yes	Yes	Yes
Skeletal muscle	Yes	Yes	Failed RNA QC	Failed RNA QC	Failed RNA QC	Failed RNA QC
Spleen	Yes	Yes	Yes	Yes	Yes	Yes
Subcutaneous fat	Yes	Yes	Failed RNA QC	Failed RNA QC	Failed RNA QC	Failed RNA QC
Testis	Yes	N/A sex-specific	Yes	N/A sex-specific	*^c^* Tissue not available	N/A sex-specific
Thyroid gland	Yes	Yes	Yes	Yes	Tissue not available	Tissue not available
Uterus	N/A sex-specific	Yes	N/A sex-specific	Yes	N/A sex-specific	Yes

These libraries were excluded from the analysis when initial clustering and quality control of the data set revealed that they did not cluster with tissue samples of the same type as expected.

These libraries were excluded from the analysis as the RIN value was very low causing a low mapping rate and high level of degradation.

This individual had been castrated at the time of tissue collection.

For RNA extraction and further downstream analysis, the beef–dairy cross (Char–Hol) tissue samples from Germany and Canadian KC cattle samples were shipped on dry ice to a central location (GIGA, University of Liège, Belgium), where the Belgian dairy (HF) tissue samples were already housed.

### RNA extraction and quality control

To minimize any batch effects, due to differences in extraction protocols across laboratories etc., RNA was extracted for all of the tissue samples at GIGA, University of Liège, Belgium. Total RNA was extracted from each tissue sample using the miRNeasy kit (QIAGEN), following the protocol provided by the manufacturer for the purification of total RNA from animal tissues. After extraction, the quantity of RNA was measured on the Nanodrop Spectrophotometer (Thermo Fisher Scientific, Waltham, MA, USA) to ensure the quantity was sufficient of CAGE sequencing. To check the quality of the RNA and detect any degradation, the RNA integrity number (RIN) was measured using the Agilent Bioanalyzer system (Agilent Technologies, Santa Clara, CA, USA). RIN values for all of the RNA samples are included in Supplementary File 1. Not all samples passed QC, the quantity of RNA was too low for CAGE library preparation from the skeletal muscle for all but the Belgian samples, and for subcutaneous fat, the quality was too low again for all but the Belgian samples. RNA samples from pancreas tissue, which is known to be high in RNases, had very low RINs. In addition, 1 mammary gland sample had a RIN of 3.4. CAGE libraries were generated for these samples, but they were later removed from the analysis (as indicated in Table 1). The number of RNA samples with a suitable quality and quantity for each population was as follows: dairy (Belgium, Holstein, *n* = 43 samples), beef–dairy cross (Charolais × Holstein, *n* = 33 samples), and composite beef (KC, *n* = 33 samples). Details of the RNA samples are included in Table 1. Aliquots containing 5 µg of total RNA were then stored at −80°C before shipping to Edinburgh Clinical Research Facility, Edinburgh, UK.

### CAGE-Seq library preparation and sequencing

CAGE libraries were prepared from 5 µg of total RNA (post DNase treatment) according to Takahashi *et al*. (2012). A modification of the original barcodes from the Takahashi *et al*. (2012) protocol (3-nt length) was required in order to perform sequencing on the Illumina NextSeq 550. This modification introduced 6-nt length barcodes for multiplexing of the libraries. The original barcodes, ACG, GAT, CTT, ATG, GTA, GCC, TAG, and TGG, were extended to a set of 21 unique 6-nt barcodes. Overall, 13 library pools were produced and sequenced on an Illumina NextSeq 550 (50-nt single end as previously described in Salavati *et al*. 2020) in 7 different runs. The details of the barcode assignments to each sample and the pool ids are described in Supplementary File 1.

### CAGE-Seq data analysis

The analysis pipeline was developed using NextFlow workflow scripting (di Tommaso *et al*. 2017). The pipeline was built using the previously described steps in https://bitbucket.org/msalavat/cagewrap_public/src/master/. After demultiplexing, trimming, and quality control, the reads were mapped against the ARS-UCD1.2_Btau5.0.1Y assembly run 9 (Hayes and Daetwyler 2019) using the nf-cage pipeline (Salavati and Espinosa-Carrasco 2022). The base pair resolution output bigWig files (2 files per sample positive and negative strand; *n* = 204 for 102 samples) were loaded in RStudio (RStudio Team 2015) (R > v4.0.0) for downstream analysis using the CAGEfightR v1.16.0 package (Thodberg *et al*. 2019).

### TSS and enhancer prediction analysis

The putative TSS and TSS-Enhancer regions were identified using the uni- and bidirectional clustering algorithms in CAGEfightR v1.16.0 as described in Thodberg *et al*. (2019). Overlapping same-strand CAGE tags mapped to either strand of the DNA were considered to be unidirectional clusters. Bidirectional TSS-Enhancer clusters were considered to be clusters of nonoverlapping tags mapped within 400–1,000 bp of each other on opposing strands (e.g. where the TSS was located on a positive strand with a nearby eRNA on the negative strand or *vice versa*). CAGE tag TSS clusters (CTSS) and their normalized expression profile [CAGE tags-per-million mapped (CTPM)] were produced using quickTSS and quickEnhancers functions of the CAGEfightR package v1.16.0. For both TSS and TSS-Enhancer regions, a minimum 10 reads per CTSS, from the entire data set, and 2/3rd sample support (i.e. if the CTSS was present in a minimum of 66/102 tissues) were imposed as filtration criteria, as previously described in Salavati *et al*. (2020). The putative regions were annotated using the assignTxID, assignTxType, assignGeneID, and assignMissingID functions of the CAGEfightR v1.16.0. The Txdb object used for annotating the CAGE-Seq data set was built using the *Bos_taurus.ARS-UCD1.2.106.gff3.gz* file from Ensembl v106.

### Mapping significant TSS and TSS-Enhancer coexpression links

Coexpression of the predicted TSS and TSS-Enhancer regions was tested using a Kendall correlation test (*P* < 0.05 sig. followed by Benjamini–Hochberg adjustment; False discovery rate, FDR < 0.01). The coexpressed pairs were identified using the findLinks function of the CAGEfightR v1.16.0 as previously described (Thodberg *et al*. 2019) and annotated using the Bos_taurus.ARS-UCD1.2 Ensembl v106 gene models. Using the gap (in bp) between the TSS (query) and Enhancer (subject) and the assigned gene symbol to either region, 3 groups of links were created: *cis* (same gene) where TSS and enhancer regions had a gap less than 1 kb; *trans* (nearby gene) where the gap was larger than 1 kb; and *novel* (cis or trans) where there was no gene annotation available for either of the linked pair. The gap size (in bp) and the Kendal correlation coefficient (range = [−1,1]) of this coexpression analysis was then used for further investigation of these links. A 2D KDE was calculated for the gap between linked TSS and enhancers versus the link's correlation coefficient. This analysis was performed using the MASS package v7.3-58.1 (Venables and Ripley 2002) (MASS::kde2d) and visualized using ggplot2 v 3.3.6 (Wickham 2009) (ggplot2::geom_density2d_filled) in R.

### Identification of long-range enhancer stretches present in the cattle genome

A hierarchical clustering of the TSS-Enhancer regions (obtained using the bidirectional analysis method in the CAGEfightR package) was performed to identify any superenhancers. Superenhancers are defined as a cluster of enhancers that occur together within a genomic region (Blobel *et al*. 2021). In this study, superenhancers were identified using a 10-kb window scan to locate stretches of the genome containing at least 3 enhancers within a window. This analysis was performed using the findStretches function of the CAGEfightR v1.16.0 followed by a Kendal correlation test of the expression matrix (CTPM values as input).

Three genomic regions harboring copy number variants (CNVs) associated with milk traits [CNV6 (chr13:70,496,054-70,623,303), CNV28 (chr7:42,700,425-42,788,788), and CNV33 (chr17:73,055,503-75,058,715)] within the cattle genome (UMD3.1) were lifted over to the ARS-UCD1.2 coordinates using the UCSC liftover tool (Hinrichs *et al*. 2006). These specific CNVs were chosen as they had been previously associated with milk production traits in an analysis by Xu *et al*. (2014). The superenhancer stretches identified in the cattle CAGE data set were overlaid with the lifted over CNV regions using IGVtools (Robinson *et al*. 2011; Thorvaldsdóttir *et al*. 2013).

### Characterizing tissue-specific TSS and TSS-Enhancers

Tissue-specific sets of TSS and TSS-Enhancers were produced in 24 separate runs of the 2 clustering algorithms (quickTSS and quickEnhancers). All samples of the same tissue type were used to create tissue-specific outputs (min 10 reads/CTSS and support 2 ≤ *n* ≤ 6). The tissue-specific TSS and TSS-Enhancer regions were also annotated using the Ensembl v106 gene models as described in the *TSS and enhancer prediction analysis* section. The expression matrix (CTPM) of all identified TSS across all tissue types was used to produce a heat map, using pheatmap v1.0.12 (Kolde 2018), based on tissue specificity indexes (TSI ranging from 0 = no expression in a particular tissue to 1 = only expressed in a particular tissue). The TSI for each TSS were produced using tspex v0.6.1 (Camargo *et al*. 2020).

### Characterizing population-specific TSS and TSS-Enhancers

Population-specific sets of TSS and TSS-Enhancers were analyzed by applying the uni- and bidirectional clustering algorithms 3 times to all tissue samples from each population of cattle: 2 Holsteins (41 samples), 2 Charolais × Holstein F2s (31 samples), and 2 KC (30 samples). In each run, only TSS and TSS-Enhancers present in all tissue types (100% support) were kept for further analysis, i.e. to define a TSS or TSS-Enhancer as Holstein specific, it had to be present in all Holstein-derived samples. A Holstein signature of TSS and TSS-Enhancers (based on start–end coordinates) was established as follows: Firstly, a set of TSS and TSS-Enhancer regions present in all 3 population sets (CHAR:KC:HOL_signature) was created, then a set shared only between HOL and Charolais × Holstein F2 sets (CHAR:HOL_signature) was created, and finally a set shared only between HOL and KC sets (KC:HOL_signature) was created. An intersection analysis was then performed using UpSetR v1.4.0 (Lex *et al*. 2014).

### Comparative analysis using the Fantom5 and sheep CAGE data sets

Mapped CAGE data sets from human (hg19, *n* = 152), rat (rn6, *n* = 13), mouse (mm9, *n* = 17), chicken (galGal5, *n* = 32), dog (canFam3, *n* = 13), and Macaque monkey (rheMac8, *n* = 15) were obtained from Bertin *et al*. (2017). The CAGE data set for sheep (PRJEB34864) (Salavati *et al*. 2020) was reanalyzed by mapping against the ARS-UI_Ramb_v2.0 (GCF_016772045.1) reference genome from NCBI v106. After remapping of these 56 ovine tissue samples, the TSS regions were annotated using the CAGEfightR v1.16.0 and GCF_016772045.1_ARS-UI_Ramb_v2.0_genomic.gff.gz gene models. The identified TSS regions and their annotated gene symbols (i.e. Ensembl attribute GENE NAME and NCBI RefSeq GENE SYMBOL) were extracted from each of the data sets for comparative analysis. TSS regions were annotated by gene symbols for all 8 data sets (in sheep and cattle using the CAGEfightR assignGeneID plugin). They were then merged based on whether the TSS region for the homologous gene symbol was shared across data sets for each species or not. This approach formed 4 distinct groups: (1) “Avian/mammalian homologs” for TSS regions shared across the data sets for all 8 species; (2) “Mammalian-specific” TSS shared across all 7 mammalian species, (3) “Human-specific” for TSS present only in human; and (4) “Species-specific” for TSS that were unique to each species. This analysis reduced the number of TSS in each data set to only those with a gene symbol annotation nearby. The majority of “species-specific” TSS for each data set had either a unique gene symbol or were novel genes with unannotated TSS regions.

### Data visualization

All data visualizations were performed in R > v4.0.0 using RStudio (RStudio Team 2015) and tidyverse suite v1.3.2 (Wickham *et al*. 2019). The nf-cage pipeline was run on the high-performance computing cluster of the University of Edinburgh (Eddie) (University of Edinburgh 2020).

## Results

### Description of CAGE libraries generated for the study

The total number of CAGE libraries generated for this study was 117, including 8 duplicated libraries to top-up the total number of reads per sample. This equated to 109 unique libraries from the tissue samples described in Table 1. From the 109 libraries, we discarded 4 pancreas samples (new total = 105), due to low RNA quality (RIN < 5) and mapping rate (<1 million reads per sample). Initial clustering of the data set revealed further 3 samples (Belgian HF female kidney and lung, and Canada KC female adrenal) that did not cluster with tissues of the same type as expected. As such, they were considered spurious and removed from the data set, giving a final total of 102 libraries. Further details of all the libraries including those that were excluded from the final analysis are included in Table 1 and Supplementary File 1.

### CAGE-Seq library size and mapping metrics

An average (±SE) of 15.5 ± 0.53 million reads per CAGE sample were generated. Details of the mapping metrics for all samples are included in Supplementary File 1. After mapping to the ARS-UCD1.2_Btau5.0.1Y (Hayes and Daetwyler 2019) reference genome, a 94% average mapping rate was achieved for all of the tissues (24 types) within the final data set [after removal of the low-quality and spurious libraries (*n* = 102)]. The tissue-specific TSS are available in Supplementary File 2.

### CAGE-Seq initial clustering and quality control

**Fig. 1.**
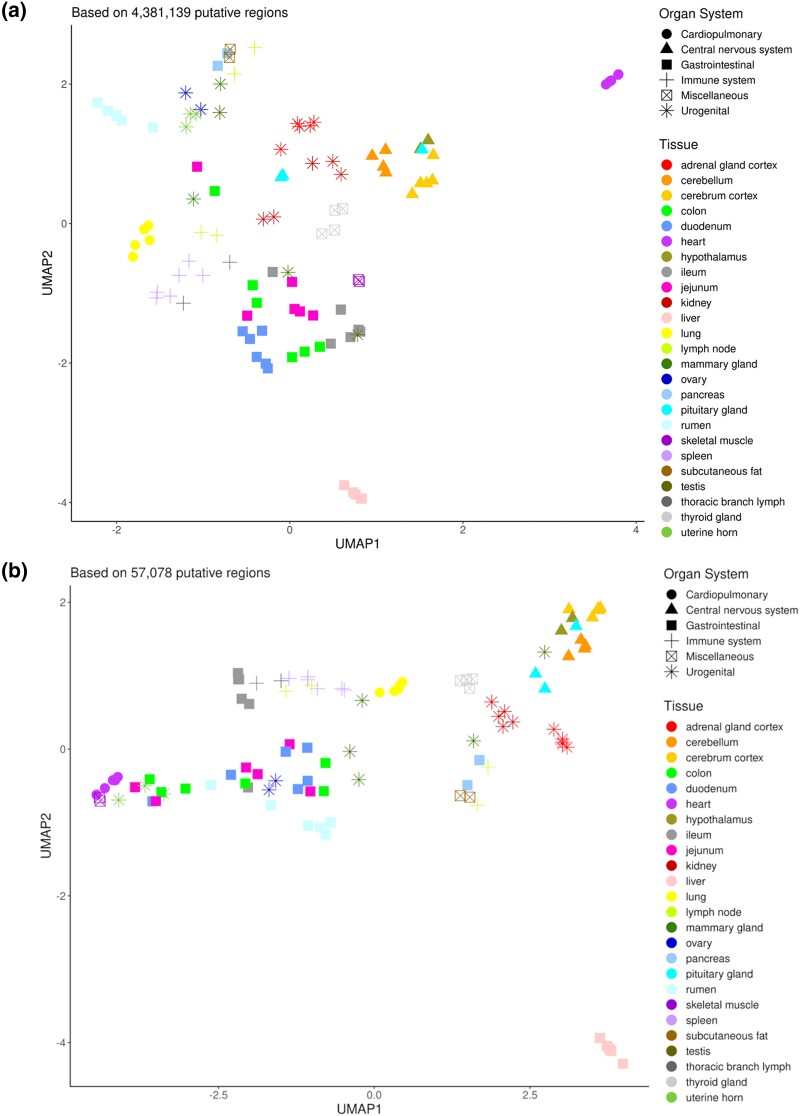
Dimension reduction of the cattle CAGE-Seq data set using uniform manifold approximation and projection (UMAP). **A**) The putative TSS (4,381,139 regions of the cattle genome) and their expression values (CTPM) for all the 102 tissue samples were used as the input matrix for UMAP. The first 2 components are visualized with tissue name (color) and organ systems (shapes) as labels. **B**) The putative TSS-Enhancers (57,078 regions of the cattle genome) and the respective CTPM values were used as the input matrix for UMAP. The first 2 components are visualized with tissue name (color) and organ systems (shapes) as labels.

After initial CAGE tag clustering (CTSS), more than 4.3 million putative TSS (unidirectional) and 57,078 TSS-Enhancer (bidirectional) regions were identified in total. A minimum of 10 reads per region was the only filtering criteria set at this stage of the analysis, with the 2/3rd tissue representation threshold being applied later. The tissue grouping of the TSS and TSS-Enhancer regions, by tissue type and grouped according to organ system, is shown in Fig. 1.

The GI tract tissues (shown as squares in Fig. 1a) and immune system tissues (lymph nodes and spleen indicated by a “+” sign in Fig. 1a) formed relatively distinct clusters as expected. Although this grouping was less pronounced in the TSS-Enhancer profiles for the immune system tissues, the GI tissues kept the original grouping structure, as shown in Fig. 1b. Specific tissues, e.g. rumen, liver, and heart, were clustered very distinctly and consistently across TSS and TSS-Enhancer profiles.

Some of the tissue samples did not cluster as expected with tissue samples of the same type from the same organ system (Fig. 1a and b). Testis, mammary gland, and pituitary gland clustered separately with an age-specific effect. This was probably due to more pronounced physiological differences in these tissues between the neonatal and adult developmental stages sampled. The lymph node tissues also separated into 2 distinct clusters which, rather than an age-specific effect, was more likely related to heterogeneity of the tissue itself and the region the sample was collected from, e.g. the cortex or the medulla. The 1 ileum sample that clustered more closely to the immune tissues was probably mistakenly collected from a Peyer's patch region of the ileum, and as such clusters more closely with the immune tissues. Any tissue samples that clustered in a highly suspect manner that could not be explained by age-specific effects or be related to the region where the tissue was sampled were removed from the final data set and not included in Fig. 1a and b (as indicated in Table 1 above).

### Identifying pervasive TSS and TSS-Enhancers across tissues

We considered a putative TSS or TSS-Enhancer region, real/reproducible only when it was present across at least 2/3rds of the tissues (Salavati *et al*. 2020). In a previous study where we performed a similar analysis for sheep (Salavati *et al*. 2020), we found after testing several thresholds that the 2/3rd tissue representation threshold was sufficiently stringent that it retained only real/reproducible TSS or TSS-Enhancer regions but not so stringent that informative regions were lost. After filtering, using the 2/3rd tissue representation threshold, 51,295 TSS and 2,328 TSS-Enhancers were detected for cattle with a mean of 91 ± 0.04 (median 94) samples supporting each putative region. Overall, 15,364 genes and 27,588 corresponding transcripts were annotated using the CAGE data set we generated for cattle. We identified 51,295 TSS regions of which 16,957 (33%) were novel and 34,338 overlapped current gene models (Ensembl v106). From the novel putative TSS regions, more than 2/3rds (67%) resided within intergenic coordinates from the ARS-UCD1.2 gene build models (Ensembl v106) and 5,592 mapped to antisense features. Complete list of the annotated TSS and TSS-Enhancers can be found in Table 2.

**Fig. 2.**
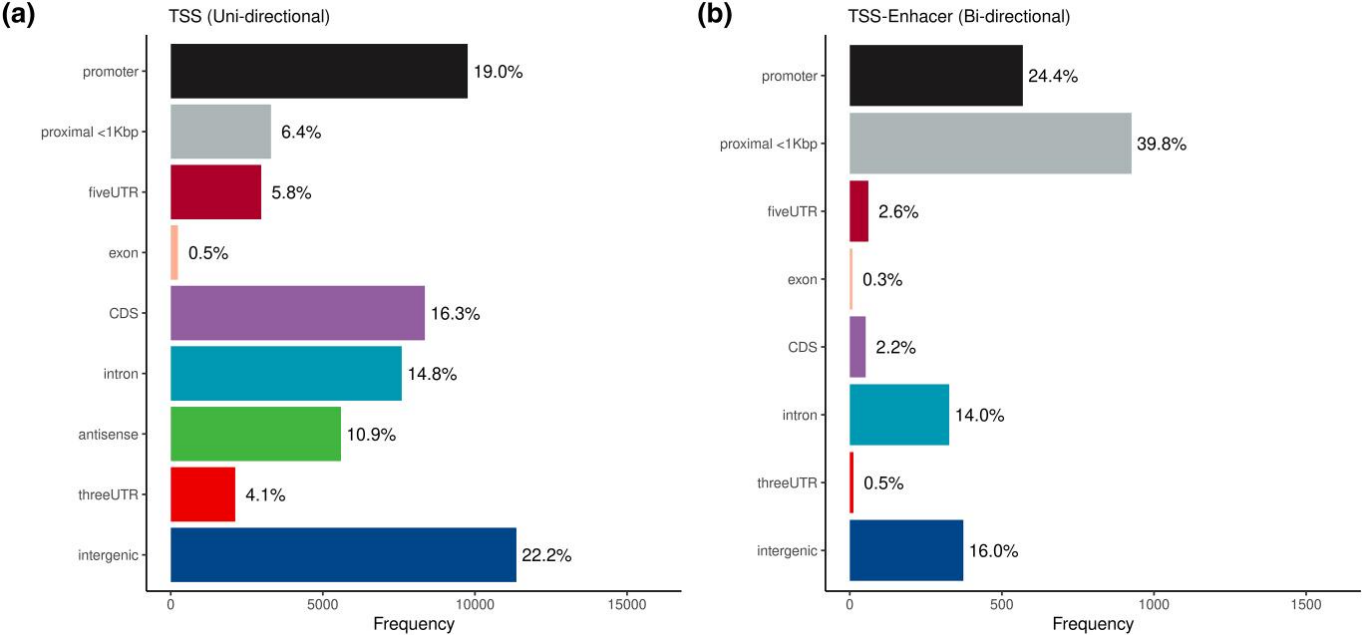
Genomic feature annotation of the cattle CAGE data set based on the Ensembl v106 annotation. **A**) Frequency distribution of the putative TSS regions identified in at least 2/3rds of the sampled tissues. **B**) Frequency distribution of the putative TSS-Enhancer regions identified in more than 2/3rds of the sampled tissues.

The median number of putative TSS regions per gene and transcript model was 1 and 2, respectively (mean 3.3 TSS/gene and 1.9 TSS/transcript). The identified TSS and TSS regions were annotated using the current Ensembl v106 gene build. Most of the annotated regions resided within the promoter and/or 1-kb proximal of the first exon. A large portion of the TSS regions (22.2%) in the data set were also located within intergenic regions (i.e. regions with no gene annotation in ARS-UCD1.2 Ensembl gff3). The frequency distribution of putative TSS regions and TSS-Enhancers based on genomic feature category are shown in Fig. 2.

### Identifying coexpressed TSS and enhancer regions

**Fig. 3.**
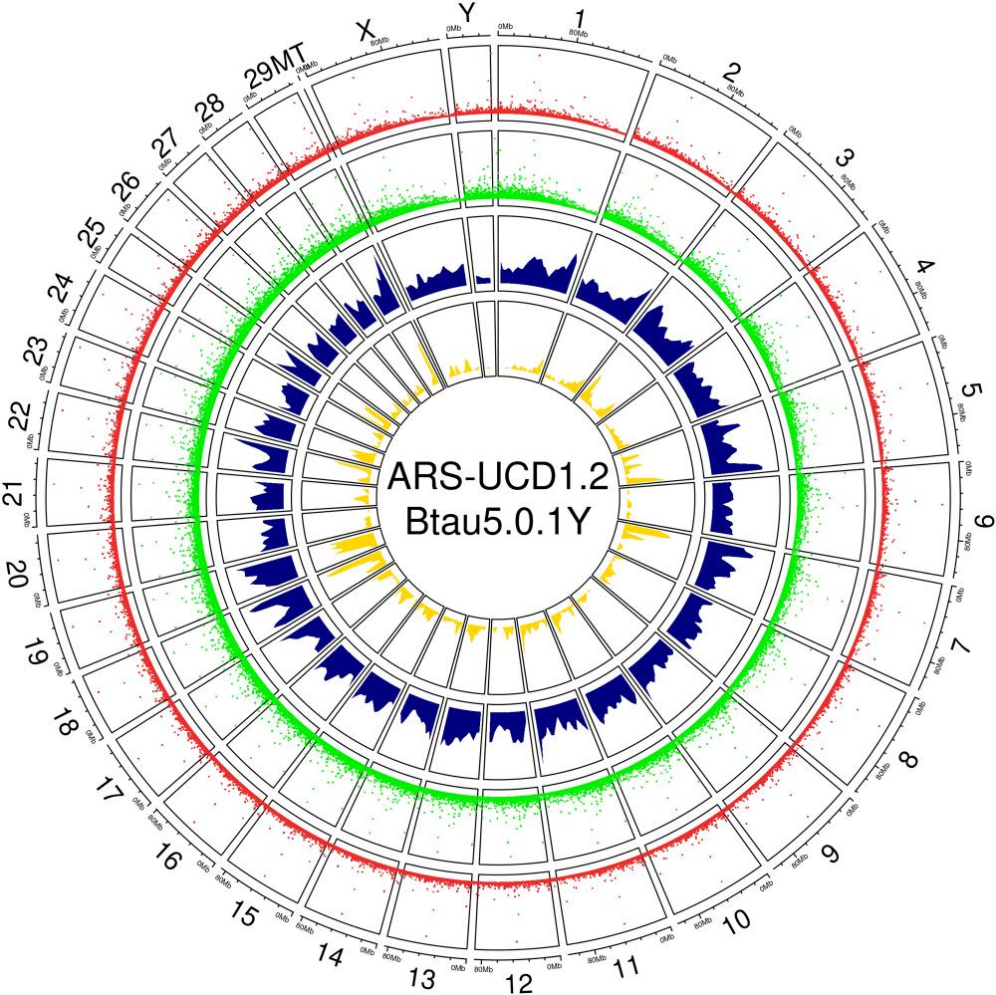
Distribution of unidirectional (TSS) and bidirectional (TSS-Enhancer) CAGE clusters within the cattle genome (ARS-UCD1.2_Btau5.0.1Y). The TSS clusters (red), TSS-Enhancer (green), significant positive (blue) and negative (yellow) correlations between coexpressed enhancer and TSS are shown as genomic tracks. The height of the tracks shows scaled expression or correlation coefficients (0–1).

In total, we identified 15,600 significant (Kendal correlation-adjusted *P* < 0.01) coexpression links between bidirectional clusters (TSS-Enhancer) and multiple unidirectional clusters (TSS). The average Kendall estimate of these coexpression links was 0.34 ± 0.001. A complete list of the coexpression links identified is provided in Supplementary File 3 (*links_df_fdr0.01_annot_KDE_input.tsv*). The expression patterns and correlation estimates are shown in Fig. 3.

**Fig. 4.**
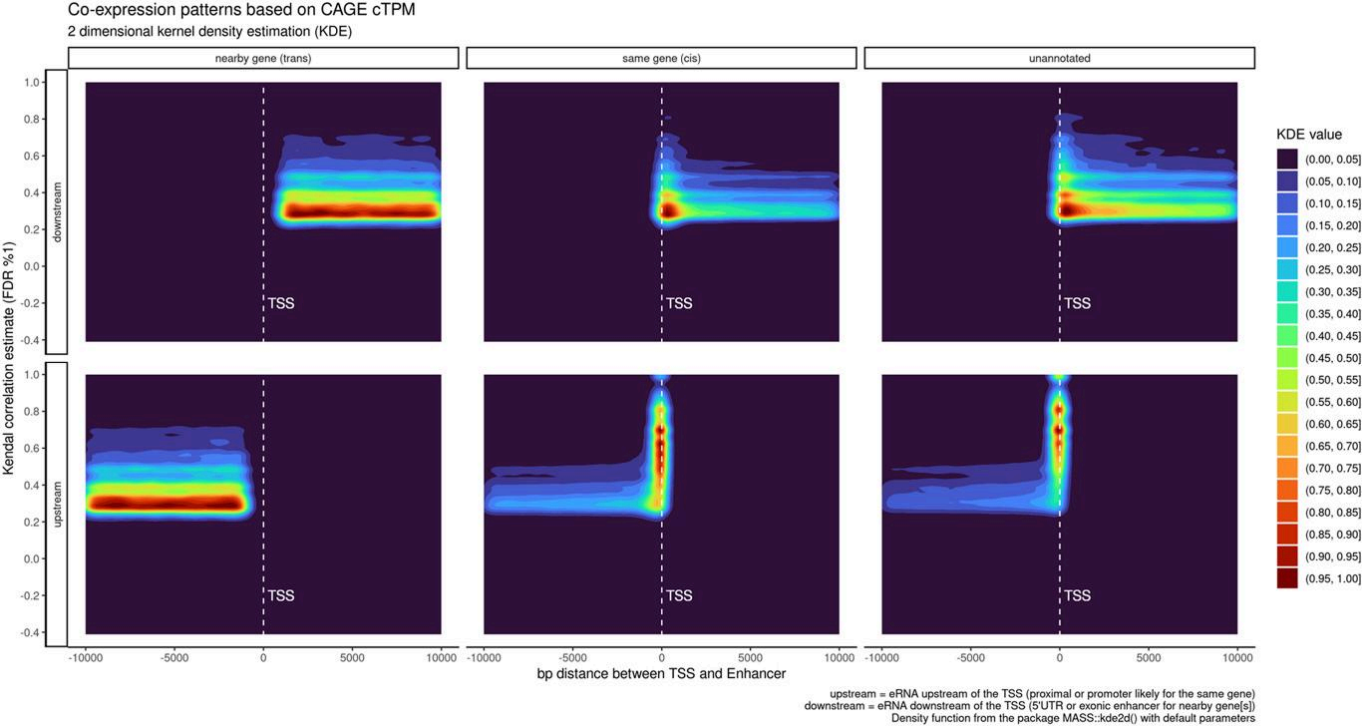
KDE of correlation coefficient (0–1) and distance to TSS (bp) of all significant coexpression profiles within the cattle CAGE data set. The Kendal correlation estimates and the distance between the enhancer region and associated TSS were used in the KDE analysis. Enhancer activity within 1 kb vicinity of the TSS was considered as the “same gene,” between 1 and 10 kb “nearby gene” while all unannotated putative TSS (termed “novel’) were linked with annotated enhancer regions marked as “unannotated.”

We further analyzed the coexpression of TSS and enhancer regions using a 2D density map. The KDE was used to identify coexpression signals based on correlation estimates versus relative distance from TSS. These signals in both annotated and unannotated putative TSS have been visualized in Fig. 4.

The KDE analysis showed a stronger coexpression (average estimate of 0.44; Welch test *P* < 0.01) for short range (<1 kb to TSS) in both upstream and downstream enhancer RNA (eRNA) compared to long range (average estimate 0.38). The longer genomic distance between TSS and coexpressed enhancers was expected to result in smaller correlation estimates. The average (up- and downstream) coexpression correlation estimate of 0.38 was with nearby genes (1- to 10-kb windows) pointing to this decay of coexpression due to the distance. Unannotated TSS and enhancer links showed the highest average correlation estimates (0.47 Welch test *P* < 0.01) compared to the other 2 categories. Further details of the comparison between groups can be found in Supplementary Fig. 1.

### Identifying long stretches of enhancer activity in the cattle genome

Analysis of the “superenhancers” (stretches of bidirectional CAGE clusters) identified 3,379 superenhancer stretches from 12,543 TSS-Enhancer clusters. The longest stretch was 54,732 bp which contained 18 TSS-Enhancers. Details of the enhancer stretches and their coordinates can be found in Supplementary File 3 (*Enhancer_stretches_10Kbp_min3_nonpervasive.tsv*). The 2/3rd tissue representation criterion was not applied to the analysis of superenhancers as they are inherently tissue specific.

We also overlaid the enhancer stretches with previously reported CNV regions of the cattle genome associated with milk production traits in Holsteins (Xu *et al*. 2014). Three milk trait-associated CNVs (chr7, ch13, and chr17 of UMD3.1 lifted to ARS-UCD1.2) had large overlaps with TSS-Enhancers identified in the following genes: *Phospholipase C Gamma 1* (*PLCG1*) (CNV at chr13:13:69,794,566-69,921,810), *Protein phosphatase 1F* (*PPM1F*) (CNV at chr17:71,988,770-71,998,055), *Topoisomerase III beta* (*TOP3B*) (CNV at chr17:71,964,684-71,967,648), and *Transport And Golgi Organization 2 Homolog* (*TANGO2*) (CNV at chr17:72,965,809-72,970,736). An example coexpression profile of TSS and enhancers for the gene *PLCG1* is shown in Supplementary Fig. 2.

### Identifying tissue-specific TSS and TSS-Enhancer regions

**Fig. 5.**
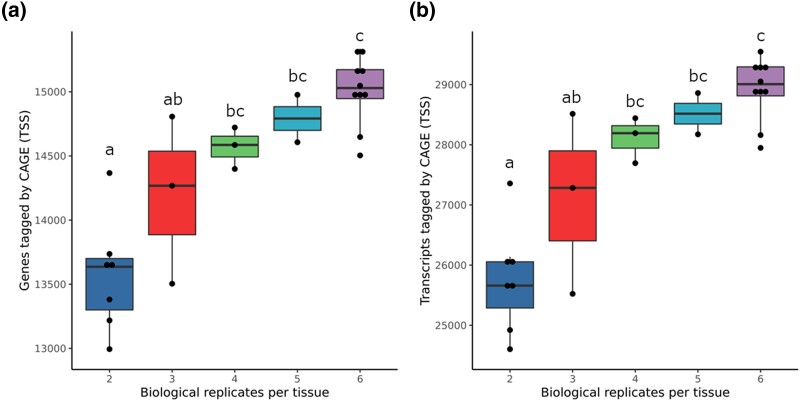
The effect of the number of replicates per tissue type and on genes **A**) and transcripts **B**) annotated by the cattle CAGE data set. All 24 tissue types were grouped by the number of biological replicates/samples previously described in Table 1. Each tissue sample is represented by a point in the above figure. The significant difference between 5 groups was tested using ANOVA followed by stats::TukeyHSD in R. The significant adjusted *P*-values are marked by letters “a,” “b,” and “c.”

Tissue-specific analysis captured, on average, 253,852 ± 24,713 (±SE) TSS clusters per tissue, 41.6% of which were novel. On average, 12,138 ± 889 TSS-Enhancer clusters per tissue were captured (27.6% novel). It was not possible to apply the 2/3rd representation threshold when identifying tissue-specific TSS and TSS-Enhancer regions, accounting for why these numbers are higher than for the previous analysis. Including multiple biological replicates per tissue type resulted in capturing a higher number of genes with CAGE tags compared to those annotated in Ensembl v106. We captured significantly (adjusted *P* < 0.05 Tukey HSD post ANOVA) less genes and transcripts annotated by CAGE tags in tissue types with 2–3 replicates compared to higher (*n* > 4) biological replicates (Fig. 5).

**Fig. 6.**
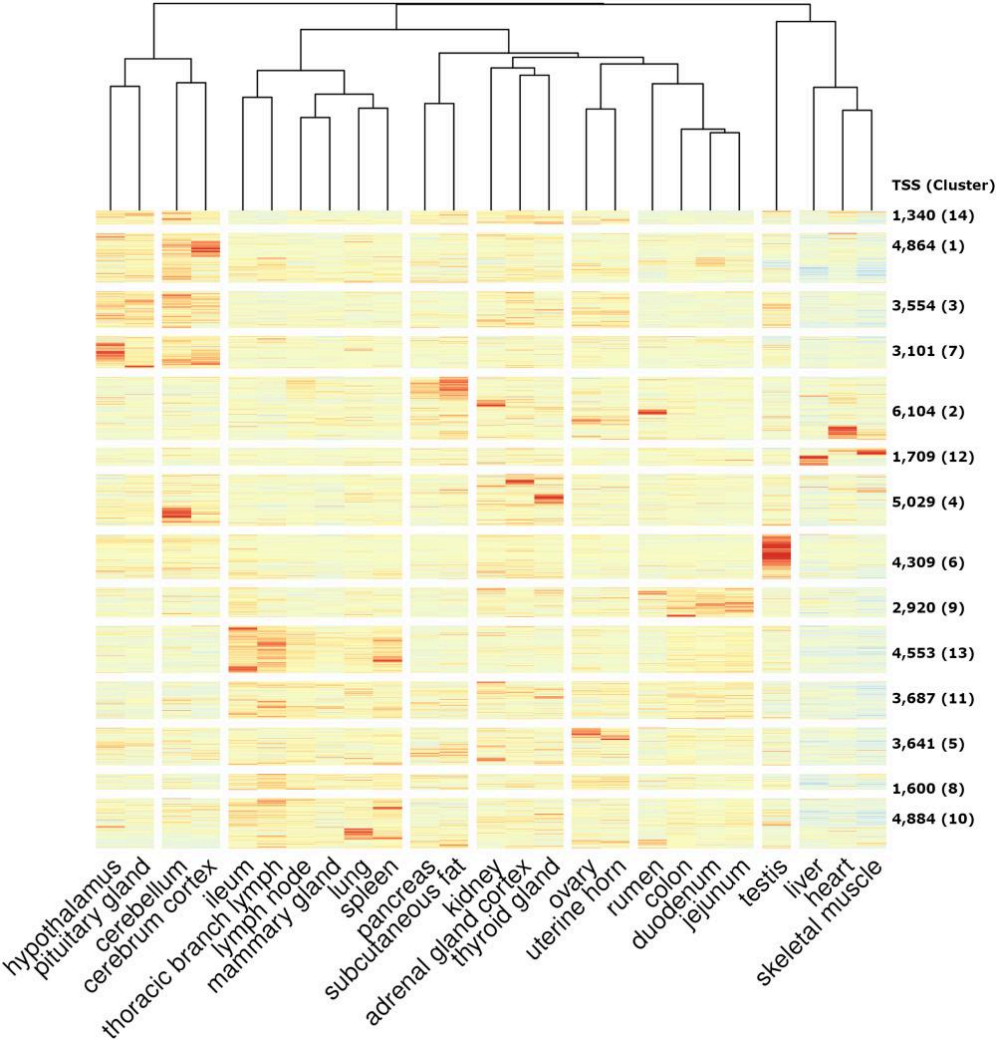
TSI for all of the putative TSS regions (*n* = 51,295) based on their CTPM in each sample. The TSI value for all samples of the same tissue (across the 3 populations) was averaged (mean) to create a single tissue-level TSI. The heatmap was generated using both row- and column-wise clustering algorithms (hclust ∼ Manhattan distances). The averaged tissue-level TSI of each TSS was center scaled and visualized as a color spectrum (lowest TSI in blue and highest TSI in red). The TSS row-wise clusters (*n* = 14) are shown on the right-hand side of the figure with the count of TSS in each cluster.

Clustering of the tissues based on the TSI (row-wise transformed CTPM) (Fig. 6) showed tissue-specific promoter activity present in testis, central nervous system tissues, GI tract, and tissues with a higher epithelial density of immune cells, e.g. ileum, mammary gland, lungs, spleen, and lymph nodes.

**Table 2.** Mapped and annotated CAGE-Seq unidirectional cluster (TSS region) cattle mapped to (ARS-UCD1.2_Btau5.0.1Y) using reference assembly gene models (using the minimum 2/3rd tissue representation threshold).

Genomic region	ARS-UCD1.2_Btau5.0.1Y		
	Novel	Annotated*^a^*	Total
Promoter Proximal 5′-UTR 3′-UTR CDS Exon Intron Antisense Intergenic	0 0 0 0 0 0 0 5,592 11,365	9,763 3,296 2,975 2,118 8,355 238 7,593 0 0	9,763 3,296 2,975 2,118 8,355 238 7,593 5,592 11,365
Total TSS Total TSS-Enhancers	16,957 373	34,338 1,955	**51,295** **2,328**
Annotated genes/transcripts			15,364/27,588

Annotated using the reference assembly gff3 track.

The numbers indicated in bold are the total number of TSS and TSS-Enhancers mapped to the cattle genome in this study.

### Population-specific TSS and TSS-Enhancer regions

**Fig. 7.**
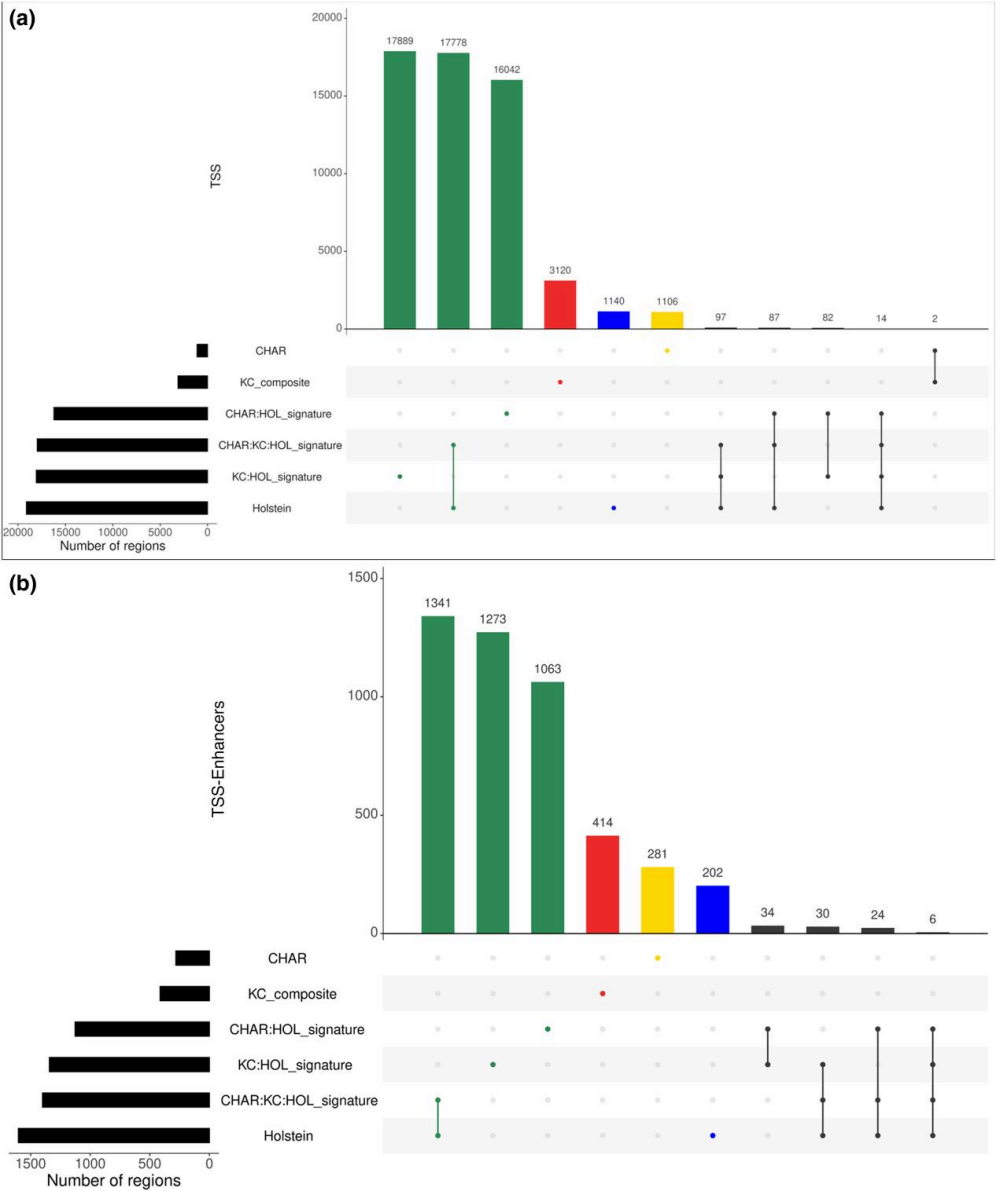
Comparative population-specific analysis of **A**) TSS and **B**) TSS-Enhancer regions across 3 populations of cattle. The intersection analysis produced 6 sets of TSS and TSS-Enhancers according to the following criteria: CHAR regions only present in tissues derived from Charolais × Holstein F2 animals, KC_composite regions only present in tissues derived from KC animals, and Holstein regions only present in tissues derived from HOL. CHAR:HOL_signature and KC:HOL_signature were regions shared between the HOL data set and 2 other populations separately. The CHAR:KC:HOL_signature was a commonly shared set of regions among all 3 populations.

Population-specific analysis showed differences in TSS coordinates and expression levels between the 3 populations of cattle (HOL, Charolais × Holstein, and KC beef cattle). The highest number of population-specific TSS was found in the KC (3,120) followed by 1,140 in Holstein and 1,106 in Charolais × Holstein. The same pattern was observed in the TSS-Enhancer regions (414 in KC, 281 in Charolais × Holstein, and 202 in Holstein). The detailed population-specific sets of TSS and TSS-Enhancer regions are provided in Supplementary File 4 and visualized in Fig. 7.

### Multispecies comparative analysis using the Fantom5 and sheep CAGE data sets

We compared the predicted TSS regions identified in the cattle CAGE data set with the previously released Ovine FAANG (Salavati *et al*. 2020) and Fantom5 CAGE data sets (Bertin *et al*. 2017). Multispecies metrics for these CAGE data sets are shown in Table 3.

**Table 3.** Comparison of the mapped TSS and annotated genes identified in other CAGE data sets (Fantom5, Ovine FAANG, and BovReg). Column “Genes’ corresponds to only the genes that were annotated using the CAGE data (using the 2/3rd tissue representation threshold). The table is sorted (in descending order) by the number of unique TSS identified in each genome.

Species	Genome	TSS↓	Genes	TSS/gene
**Human** **Mouse** **Cattle** **Chicken** **Rat** **Sheep** **Sheep** **Rhesus monkey** **Dog**	hg38 mm10 **ARS-UCD1.2_Btau5.0.1Y***^a^* galGal5 rn6 **Oar rambouillet v1.0***^b^* **ARS-UI_Ramb_v2.0***^c^* rheMac8 canFam3	209,911 164,672 **51,295** 32,015 28,497 **28,148** **27,011** 25,869 23,147	31,184 30,501 **15,364** 7,759 13,719 **13,912** **13,771** 8,047 5,288	6.7 5.4 3.3 4.1 2.1 2 2 3.2 4.4

Ensembl gff3 annotation v106 track lifted over to 1000 Bull Reference Genome.

NCBI RefSeq gff3 annotation v100.

NCBI RefSeq gff3 annotation v104.

The numbers indicated in bold are the total number of TSS and TSS-Enhancers mapped to the cattle genome in this study.

**Fig. 8.**
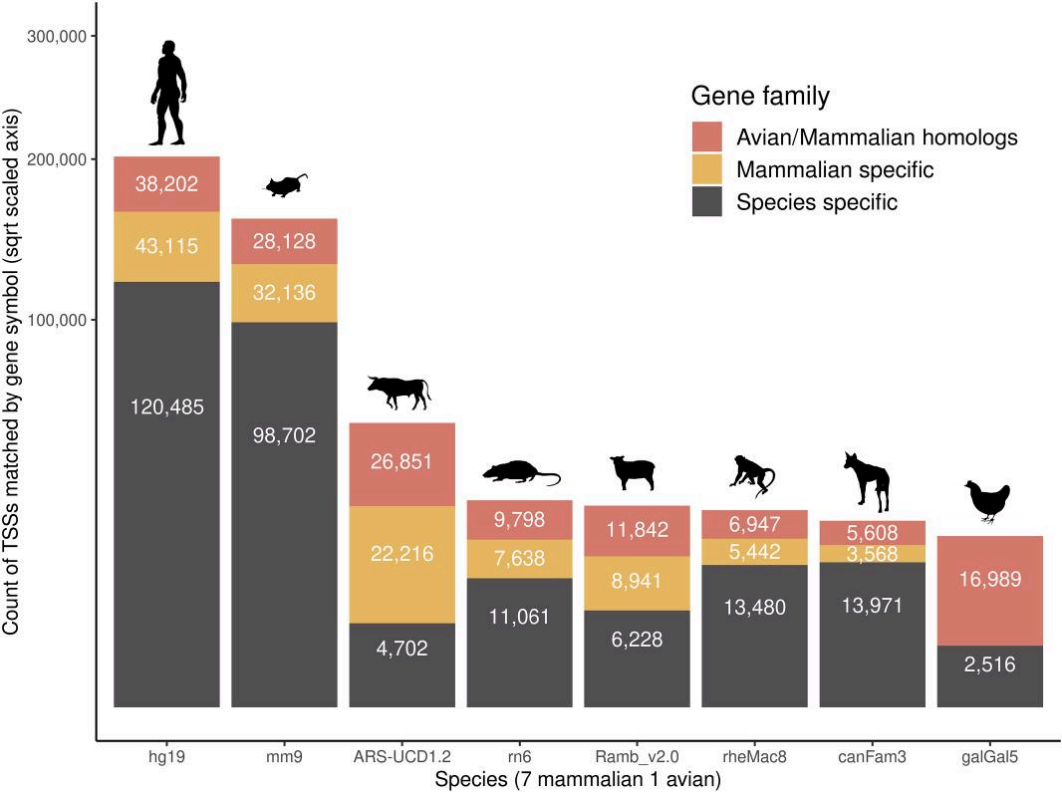
Distribution of the annotated TSS regions (gene symbols) across 8 species. The Fantom5 human, mouse, rat, dog, rhesus monkey, and chicken CAGE predicted promoter regions were analyzed and compared with the cattle and sheep annotated data sets. The TSS regions, annotated by gene symbols, were colored in each data set based on avian/mammalian origin (gene symbols present in all 8 species), mammalian specific (7 mammalian species), human or species specific (gene symbol unique to human or each species).

Remapping of the CAGE data set for sheep to the current ARS-UI_Ramb_v2.0 reference genome assembly when compared with the previous version Oar_rambouillet_v1.0 slightly reduced the number of TSS identified in the sheep CAGE data set (∼5% less TSS and ∼2% less annotated genes). A comparison of the CAGE (TSS) annotated genes from different avian and mammalian species showed high levels of overlap with both cattle and the remapped sheep CAGE data set. Overall, we were able to identify 4,702 genes and their associated TSS unique to the cattle genome (Fig. 8).

## Discussion

High-resolution mapping of the actively transcribed regions of the genome can help to identify the drivers of gene expression, regulation, and phenotypic plasticity (Tippens *et al*. 2018). Defining TSS within promoter regions can provide information about how genes controlling traits of interest are expressed and regulated. In this study, we used CAGE sequencing of 24 tissue types from 2 individuals from 3 different populations of cattle to improve the annotation of TSS and enhancers in the current reference genome for cattle (ARS-UCD1.2). We identified more than 51k unique putative TSS coordinates (22% in unannotated regions of the cattle genome). This data set provides a high confidence set of promoter annotations for the cattle transcriptome including “novel” promoters not previously annotated in the available NCBI v.106 and Ensembl v.106 annotations (25% of TSS overlapped with currently annotated promoters and were 1 < kb proximal to annotated gene models).

Similar to previously reported studies in cattle (Goszczynski *et al*. 2021), pig (Halstead *et al*. 2020; Kern *et al*. 2021), and human (Andersson *et al*. 2014), we also identified both tissue- and population-specific sets of TSS and TSS-Enhancers. Recently, new genomic resources have been generated for farmed animal species, including pangenomes and breed-specific reference quality assemblies (e.g. Li *et al*. 2019; Crysnanto *et al*. 2021; Talenti *et al.* 2022). Usage of breed-specific genome assemblies can provide a more accurate picture of structural variants specific to a population of animals and ensure better mappability for sequence data in reference-guided approaches. Identifying breed-, population-, or species-specific promoter complexity can help to harness the full potential of these assemblies as tools to inform genomics-enabled breeding programs, e.g. reviewed in Georges *et al*. (2019) and Clark *et al*. (2020). We identified full tissue support for TSS and TSS-Enhancer regions unique to each of the 3 populations of cattle in this data set. This finding further highlights the value of including samples from more than 1 breed in creating reference annotation data sets. The highest number of TSS and TSS-Enhancer regions was present in the most diverse population (KC). However, for the comparative population-specific analysis, it should be noted that the animals from the 3 populations were each of different ages. As such, separating a population effect from an age effect is difficult and should be considered as a limitation of the study.

Using methodology for identifying longer stretches of superenhancers (Thodberg *et al*. 2019) identified 3,379 superenhancer stretches from 12,543 TSS-Enhancer clusters. The longest stretch was 54,732 bp which contained 18 TSS-Enhancers. This number is comparable with other studies, e.g. Zhang *et al*. (2022) identified several hundred genes linked to superenhancers in pig, mouse, and human using ChIP-sequencing (chromatin immunoprecipitation). Comparative analysis of the CAGE data set with ChIP-seq data generated for the same tissues, which will soon be available for the BovReg project (Moreira *et al*. 2022), should validate the results obtained in this study.

As an example of how to link the CAGE data sets to traits of interest, we overlaid the superenhancer regions with information for CNVs associated with milk yield traits in cattle (Xu *et al*. 2014). This analysis revealed CNVs that had large overlaps with superenhancers in genes *TOP3B* and *PPM1F*, which had no obvious associations with phenotypic traits in cattle beyond those identified by Xu *et al*. (2014) for milk production traits. However, a CNV-associated superenhancer region was identified for the gene *PLCG1*, which was reported as a stature (chest width) phenotype-associated QTL target in Simmental (dual purpose) cattle by Doyle *et al*. (2020). *PLCG1* has also been reported as a differentially expressed gene between high/low gain versus high/low intake among *n* = 143 cross-bred steers from 15 different beef breeds by Zarek *et al*. (2017). In addition, the expression of *PLCG1* has been shown to be downregulated due to maternal under nutrition in the muscle tissues of Japanese Black calves raised on a low nutritional value diet (Muroya *et al*. 2021). Given the critical role of *PLCG1* in both muscle growth and metabolism in beef cattle, the knowledge of its associated superenhancer coordinates and coexpressed promoter regions across tissues could serve as a guide for future functional validation, gene editing, or marker selection studies. Another CNV-associated superenhancer region identified in our data set was *TANGO2*, a Golgi system-associated protein coding gene mainly associated with mitochondrial disease (Heiman *et al*. 2022). *TANGO2* has been shown to be overexpressed in seminal plasma of lowly/subfertile bulls (Muhammad Aslam *et al*. 2014) and is highly associated with multiple heifer fertility traits in the Holstein cattle population (Chen *et al*. 2022). Knowledge of the regulatory landscape of genes such as *TANGO2* provides a path for understanding the role of these genes in cattle fertility phenotypes.

We also compared cattle data sets with other publicly available TSS and TSS-Enhancer genomic tracks for sheep and other mammalian and avian species to further identify promoters specific to the cattle genome. Using a homolog-matching approach, the TSS annotation of the cattle data set captured the highest number of mammalian and (or) avian gene families represented in the data sets, after human and mouse, demonstrating how comprehensive the data set generated for cattle is. Such information could be used to understand how the genome controls traits in different species and to identify regions that are important for conservation in breeding programs. However, merging based on shared gene symbols is a basic comparison that does not consider scenarios such as gene paralogs and multiple accepted gene symbols. As such, it is likely that our analysis might miss certain mammalian-specific or avian–mammalian shared genes and mistakenly attribute them as species specific. This should be considered a limitation of the study.

Future work will integrate the CAGE data set we have generated for this study with other omics data sets. These include ChIP-Seq and ATAC-Seq (Assay for transposase-accessible chromatin), generated for the same tissue samples, as part of additional efforts to annotate the cattle genome for the BovReg project (Moreira *et al*. 2022). This comparative analysis will contribute to a better understanding of the function of regulatory variants, such as those identified by genome-wide association studies, improving our knowledge of the genomic control of complex traits in cattle. In addition, the CAGE data produced for this study will be combined with transcriptomic data sets (mRNA, miRNA, and total RNA-Seq) produced by BovReg partners. This will provide a new comprehensive transcriptome annotation for the cattle genome (ARS-UCD1.2), as a resource for the farmed animal genomics community.

## Data Availability

The raw sequence data for all the CAGE-Seq libraries are available via the European Nucleotide Archive under BioProject ID PRJEB43235 and via links in the FAANG Data Portal for the BovReg Project (https://data.faang.org/projects/BovReg). The tissue-level TSS and TSS-Enhancer region tracks are also available via the genome browser for the FAANG data portal (https://api.faang.org/files/trackhubs/BOVREG_CAGE_EUROFAANG/). The tissue- and population-specific sets of TSS and TSS-Enhancer predictions are provided in Supplementary Files 2 (supplementary file2.zip) and File 4 (supplementary file4.zip), respectively, which can be downloaded from FigShare via this link: https://doi.org/10.6084/m9.figshare.21769649. The code and documented analysis pipeline developed in NextFlow DSL2 syntax (di Tommaso *et al*. 2017) is available at https://github.com/mazdax/nf-cage. All the supplementary files and figures associated with this publication are available via the following link: https://doi.org/10.6084/m9.figshare.21769649.
